# Effectiveness of Influenza Vaccines and Duration of Protection Against Hospitalisation During the 2024/25 Season in Northern Ireland, UK

**DOI:** 10.1111/irv.70303

**Published:** 2026-08-06

**Authors:** Magda Bucholc, Siobhán Murphy, Mark G. O'Doherty, Suzanne Wilton, Emma Dickson, Declan T. Bradley

**Affiliations:** ^1^ Public Health Agency Belfast UK; ^2^ Centre for Public Health Queen's University Belfast Belfast UK

**Keywords:** hospital admission, hospitalisation, influenza, influenza A, influenza B, vaccine, vaccine effectiveness, vaccine type

## Abstract

**Background:**

Seasonal influenza causes substantial morbidity and hospitalisation each year. We estimated influenza vaccine effectiveness (VE) against laboratory‐confirmed influenza‐associated hospitalisation among patients in Northern Ireland (NI) during the 2024/25 influenza season.

**Methods:**

We used a test‐negative design to estimate VE against hospitalisation. Influenza‐positive cases and test‐negative controls were identified through the national laboratory surveillance system and linked to hospital admission and vaccination records. VE was estimated by influenza type/subtype, age group, sex, vaccine type and time since vaccination.

**Results:**

Among 15,133 hospitalised patients, 2024 (13.4%) tested positive for influenza. Among the 2024 patients admitted with laboratory‐confirmed influenza, the vast majority tested positive for influenza A (*n* = 1803; 89.1%), whereas 221 cases (10.9%) were influenza B. Subtyping of influenza A identified 606 A(H1) infections and 93 A(H3) infections; the remaining 1104 influenza A samples were not subtyped. VE against laboratory‐confirmed influenza infection was 46.0% (95% CI: 39.7% to 51.8%), with higher VE in children aged 2–17 years (60.8%; 95% CI: 48.3% to 70.5%) than in adults aged 18–64 years (40.5%; 95% CI: 24.9% to 53.2%) and ≥ 65 years (42.3%; 95% CI: 33.2% to 50.1%). VE against influenza A across all ages was 41.4% (95% CI: 34.2% to 47.9%). Vaccination reduced the odds of hospitalisation due to influenza A(H1) by 44.3% (95% CI: 33.2% to 53.7%) and A(H3) by 49.9% (95% CI: 20.3% to 69.3%). VE against influenza B was higher at 76.4% (95% CI: 64.9% to 84.7%). For influenza A, VE was highest 2–8 weeks after vaccination at 51.9% (95% CI: 42.1% to 60.1%) and declined with time since vaccination to 44.6% (95% CI: 35.6% to 52.5%) at 9–16 weeks and 41.4% (95% CI: 15.4% to 60.1%) at ≥ 16 weeks. VE against influenza B remained high throughout the season. No statistically significant differences in VE by vaccine type were found.

**Conclusions:**

Influenza vaccination reduced the risk of hospitalisation with laboratory‐confirmed influenza during the 2024/25 season, offering meaningful protection at individual and population levels, with the greatest benefit observed in children.

## 1 Introduction

Seasonal influenza remains a major cause of severe respiratory illness and death worldwide [1]. In Europe, annual epidemics infect a substantial proportion of the population and are associated with considerable excess mortality, the majority of which occurs in people aged 65 years and older [2].

Following atypical circulation during the COVID‐19 pandemic, influenza activity largely returned to pre‐pandemic patterns in the 2023/24 season, although with marked variation in timing and intensity across countries [3]. As COVID‐19‐related morbidity declines, influenza has re‐emerged as a key driver of winter hospital admissions and health system pressure, particularly among older and clinically vulnerable individuals.

Annual influenza vaccination is the primary strategy for preventing severe disease. However, vaccine effectiveness (VE) varies across seasons and population groups, influenced by viral evolution, vaccine composition, host factors and waning immunity over time [4, 5]. Antibody responses peak shortly after vaccination but decline over subsequent months, raising concerns about reduced protection later in the season [4]. High‐quality, real‐world estimates of VE are therefore essential to inform vaccination policy and optimise programme impact.

During the 2024/25 influenza season, interim VE has been evaluated across multiple settings and populations [6, 7, 8, 9, 10, 11]. An interim European multi‐country study, comprising eight studies across 17 countries, reported interim VE estimates against any laboratory‐confirmed influenza ranging from 40% (95% CI: 26%–52%) to 53% (95% CI: 47%–58%) in primary care settings and from 34% (95% CI: 28%–39%) to 60% (95% CI: 39%–74%) in hospital settings across all age groups [8]. In the United States, VE against outpatient influenza (across outpatient clinics, urgent care clinics and emergency departments) ranged from 32% to 60% among children and adolescents aged under 18 years, with higher protection against influenza‐associated hospitalisation (63%–78%) [9]. Among adults, VE estimates were generally lower [9]. In Germany, Erdwiens et al. [10] reported interim VE against any influenza at 31% (95% CI: 1%–52%) in primary care and 69% (95% CI: 21%–88%) in secondary care. Differences in VE by vaccine formulation have also been observed [7]. A Danish registry study among adults aged 65 years and older found higher VE for adjuvanted and high‐dose quadrivalent influenza vaccines (48%; 95% CI: 42%–52% and 50%; 95% CI: 38%–59% respectively) compared with standard‐dose formulations (33%; 95% CI: 24%–41%) [7].

In Northern Ireland (NI), influenza surveillance data for the 2024/25 season showed a sustained rise in activity beginning in mid‐to‐late November 2024 [12]. Transmission intensified through December, reaching a seasonal high towards the end of that month, before declining in early 2025. Although influenza activity decreased after December, increased circulation of influenza B from January 2025 resulted in a slower overall decline in influenza cases compared with the previous season. During the 2024/25 influenza season, influenza A(H1) was the most prevalent subtype, with higher circulation than influenza A(H3). Overall indicators of activity demonstrated a more intense influenza season than the 2023/24 season.

Influenza testing in hospitals was undertaken as part of routine clinical care. All hospital in‐patients with symptoms of respiratory infection were tested for influenza A and B using molecular (RT‐PCR) assays. Additionally, point of care molecular testing for influenza was available in some emergency departments.

Delivery of the 2024/25 influenza immunisation programme in NI was undertaken through a range of healthcare settings, including GP practices, community pharmacies, Health and Social Care (HSC) Trusts and school nursing services. Vaccination for most eligible groups commenced on 7 October 2024, aligning with national guidance to optimise protection during peak winter transmission. However, the school‐based programme and vaccination of pregnant women began earlier, from 13 September 2024, once vaccine stock became available.

Eligibility criteria followed national recommendations and prioritised those at elevated risk of severe influenza outcomes. Groups offered vaccination included children aged 2–17 years; adults aged ≥ 65 years (with eligibility extended to those aged 50–64 years from January 2025); individuals aged 18–64 years in clinical risk groups; at‐risk children aged 6 months to under 2 years; pregnant women; carers and close household contacts aged 18–64 years; and HSC workers [13, 14]. The school‐based influenza vaccination programme offered vaccination to all children attending primary school (Years 1–7), special school and post‐primary school (Years 8–12) during the 2024/25 academic year [13]. The offer of vaccination was extended to include all those aged 50–64 years to make use of available purchased influenza vaccine stock that would not have been otherwise used [15]. In line with the recommendation in Chapter 19 of the Greenbook: Influenza to offer seasonal influenza vaccination to those at higher risk of infection with avian influenza related to their work or similar exposures, the programme was further extended on 5 December 2024 to include high‐risk poultry and avian‐exposed workers [16]. Adults aged ≥ 65 years were primarily offered the adjuvanted quadrivalent inactivated vaccine (aQIV). The quadrivalent cell‐based vaccine (QIVc) was recommended for adults aged 18–64 years in clinical risk groups, pregnant women, carers, close contacts of immunocompromised individuals and healthcare workers and was additionally offered to those aged 50–64 years from January 2025. Children aged 2–17 years received the live attenuated influenza vaccine (LAIV) unless contraindicated, in which case QIVc was provided. QIVc was also administered to at‐risk children aged 6 months to under 2 years. Across the season, 593,318 influenza vaccine doses were delivered. Uptake among adults was highest in care home residents (80%) and those aged ≥ 65 years (73.7%). Among children, the highest uptake was observed in primary school children (64.9%) [17].

The primary objective of this study was to estimate influenza VE against influenza‐associated hospitalisation and to examine the duration of protection during the 2024/25 respiratory virus season in NI.

## 2 Methods

### 2.1 Study Design and Population

We undertook a retrospective observational study using a test‐negative case–control design (TND) to estimate the end‐of‐season effectiveness of the influenza vaccine in preventing hospitalisation due to laboratory‐confirmed influenza infection during the 2024/25 season in NI.

The study population included all community‐acquired hospital admissions for individuals aged 2 years and older who tested for influenza using RT‐PCR during the influenza season. Influenza testing was undertaken as part of routine clinician‐directed diagnostic care for patients with clinically suspected acute respiratory infection. Testing was performed when clinically indicated to support diagnosis, patient management, antiviral treatment, or infection prevention and control. The operational datasets available through the Public Health Agency's Memorandum of Understanding with HSC Trusts did not include presenting symptoms or ICD‐10‐coded admission diagnoses.

Admissions were classified as community‐acquired when respiratory swabs were collected within 14 days prior to admission or within 2 days following admission and for which laboratory test results were available in the laboratory‐based regional surveillance system. Admissions with respiratory specimens collected more than 2 days after admission were considered potentially hospital‐acquired and were excluded. Individuals were also excluded if the swab collection date was unknown or fell outside the study period, or if influenza vaccination occurred within 0–13 days prior to swab collection.

Patients with a positive influenza A or influenza B RT‐PCR result were classified as cases. Patients testing negative for influenza were classified as controls. SARS‐CoV‐2‐positive patients were excluded from the primary comparison group to reduce bias arising from exposure risk or vaccination status.

### 2.2 Study Period

The study period corresponded to epidemiological week 40 of 2024 (beginning 30 September 2024) through week 15 of 2025 (ending 12 April 2025). This period encompasses the main period of influenza circulation in NI, coincides with the timing of the seasonal influenza vaccination programme and broadly aligns with periods commonly used in end‐of‐season influenza VE studies elsewhere in Europe. Although a small number of influenza cases occurred outside this timeframe, the study period captured the vast majority of seasonal influenza activity observed during the 2024/25 season.

### 2.3 Exposure and Outcome Definitions

Individuals were classified as vaccinated if they received a 2024/25 influenza vaccine at least 14 days prior to testing; those without a recorded vaccine were considered unvaccinated. Individuals tested within 14 days of vaccination (*n* = 365) were removed from the primary analysis to allow for maturation of post‐vaccination immune responses. The study outcome was laboratory‐confirmed influenza identified by RT‐PCR testing among all‐cause hospitalised patients meeting the study inclusion criteria. Information on admission diagnoses and ICD‐10 coding was not available; therefore, analyses could not be restricted to acute respiratory infection or influenza‐like illness admissions.

### 2.4 Laboratory Testing

All hospital in‐patients with symptoms of respiratory infection were tested for influenza A and B using RT‐PCR assays. Additionally, point of care molecular testing for influenza was available in some emergency departments. Given the winter respiratory season, testing for influenza followed a tiered approach. Urgent samples were processed in local Trust microbiology laboratories using the GeneXpert (Cepheid) platform. Routine samples from hospitalised patients were referred to the Regional Virus Laboratory, where they were tested for influenza A and B, SARS CoV 2 and respiratory syncytial virus (RSV) by RT PCR on the high throughput COBAS 8800 (Roche). This testing combined a commercial SARS CoV 2 assay with a laboratory developed multiplex PCR assay for influenza A and B and RSV.

Influenza‐positive specimens were classified as influenza A or influenza B. Influenza A viruses were further characterised as A(H1), A(H3), or untyped where subtyping for influenza A results were unavailable. Full neuraminidase subtyping and influenza B lineage assignment were not routinely undertaken.

### 2.5 Data Sources

To construct the analytic dataset, laboratory results, vaccination information and hospital administrative records were linked deterministically using a pseudonymised NI Health and Care Number (HCN). Data were sourced through routine surveillance systems. Vaccination information was obtained from the NI Vaccine Management System (VMS), the official system of record for vaccination programmes delivered across Northern Ireland. The VMS captures vaccination data from all eligible individuals vaccinated through general practices, community pharmacies and school‐based vaccination programmes. Influenza vaccinations delivered through school‐based programmes are initially recorded in the NI Child Health System and subsequently transferred to the VMS. Individuals without a record of receipt of a 2024/25 influenza vaccine in the VMS were classified as unvaccinated. Influenza RT‐PCR results were provided by the NI Regional Virus Laboratory and HSC Trust point‐of‐care testing. Hospital admission data were obtained from the five acute HSC Trusts (Belfast, South Eastern, Southern, Northern and Western), which collectively provide secondary care services for the entire NI population.

### 2.6 Statistical Analysis

VE was derived from adjusted odds ratios (aOR) estimated using multivariable logistic regression models comparing vaccination status between cases and controls. Candidate covariates were selected a priori based on epidemiological relevance and included age, sex, epidemiological week (modelled using spline terms) and HSC Trust. VE was computed as (1 − aOR) × 100%.

Subgroup analyses explored VE by influenza type and subtype, predefined age groups (2–17, 18–64, 65+), sex, time since vaccination and vaccine type. To assess whether VE differed across these subgroups, we evaluated effect modification by including an interaction term between vaccination status and the stratifying variable (age group, sex, vaccine type) in logistic regression models. Information on underlying medical conditions was not available within the linked datasets and therefore could not be included as an adjustment variable. For time since vaccination, we examined waning of VE by comparing odds of infection across defined intervals since vaccination: for influenza A, 2–8 weeks, 9–16 weeks and > 16 weeks; for influenza B, 2–16 weeks and > 16 weeks. For influenza B, broader intervals were used because influenza B activity increased later in the season, beginning in January, resulting in fewer cases earlier in the season and limited statistical power to support finer stratification while maintaining stable VE estimates. Pairwise comparisons of VE across subgroups were conducted using estimated marginal means. All statistical analyses were conducted in R version 4.4.0. All statistical analyses were conducted in R version 4.4.0, using the splines, stats, broom, dplyr, emmeans and logistf packages.

### 2.7 Sensitivity Analyses

We undertook four sensitivity analyses. First, we incorporated patients categorised as partially vaccinated, i.e., those who received an influenza vaccine 7–14 days before specimen collection—to assess whether evidence for early protection can be detected. Second, individuals who tested positive for SARS‐CoV‐2 were included as controls to evaluate whether their inclusion bias VE estimates, as suggested in TND studies due to correlated vaccination behaviour [18]. Third, to mitigate potential bias in subgroups with limited case numbers (e.g., influenza A(H1), A(H3), B), we re‐calculated VE using Firth's penalised logistic regression. Finally, VE was re‐estimated restricting the analysis to emergency admissions only, to examine whether vaccine performance differed in the subset of cases most likely to represent acute, community‐acquired illness.

## 3 Results

### 3.1 Study Population

**FIGURE 1:**
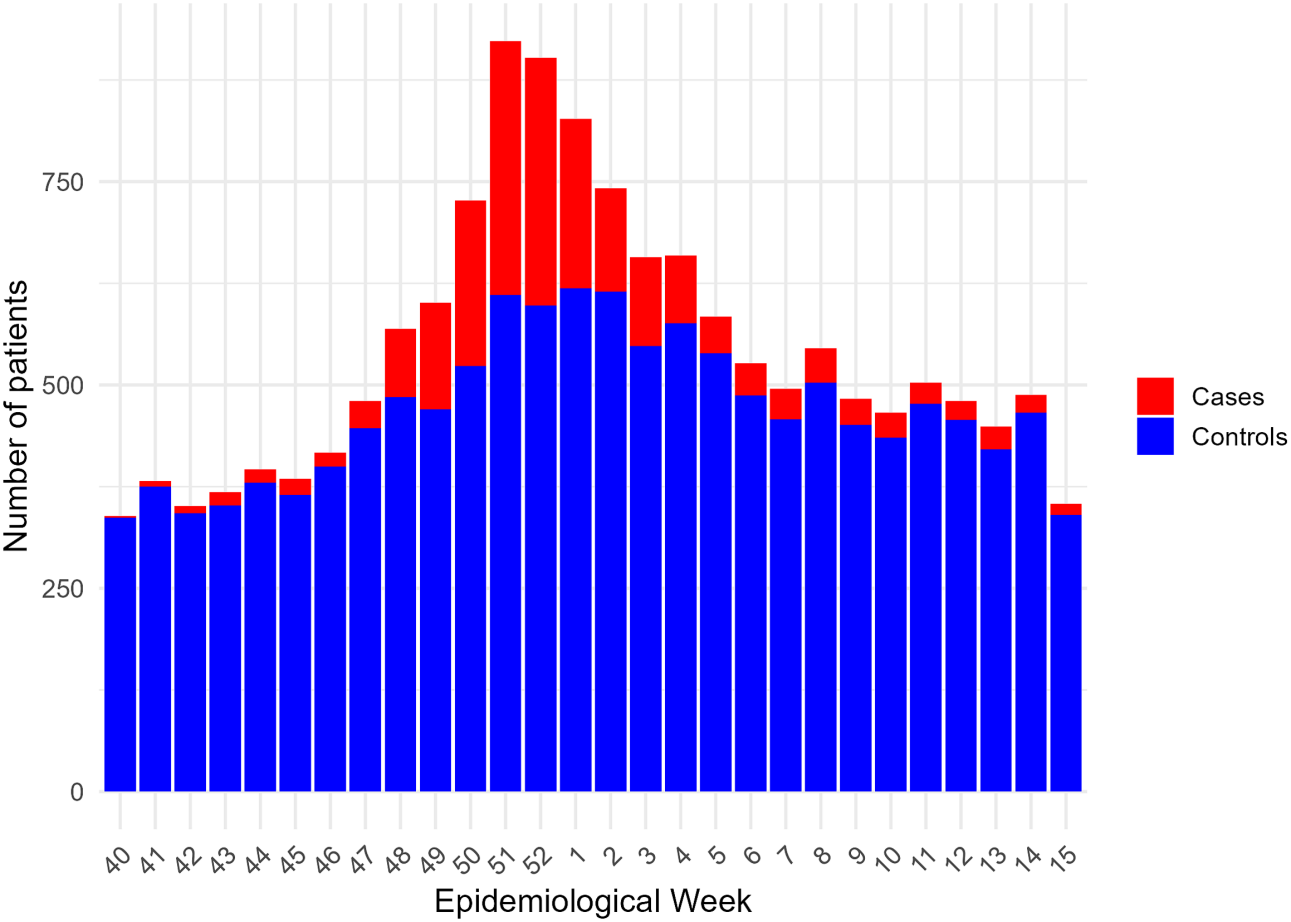
Weekly number of influenza cases and test‐negative controls included in the study. Data are shown by epidemiological week of sample collection from week 40 of 2024 (beginning 30 September 2024) through epidemiological week 15 of 2025 (ending 12 April 2025).

In total, 15,133 hospitalised individuals were included in the study, of whom 2024 (13.4%) tested positive for influenza (Table 1). Influenza A accounted for the majority of confirmed cases (*n* = 1803; 89.1%) whereas influenza B represented 221 cases (10.9%). Subtyping of influenza A samples identified 606 A(H1) and 93 A(H3) infections; the remaining 1104 influenza A samples were not subtyped. Vaccination coverage differed between cases and controls: 41.7% (*n* = 5471) of influenza‐negative controls were vaccinated, compared with 31.7% (*n* = 641) of all influenza‐positive cases. Among influenza A cases, 34% (*n* = 613) had been vaccinated, whereas the proportion of vaccinated individuals among influenza B cases was lower at 12.7% (*n* = 28). The median age of all influenza cases was 63 (IQR: 34–79) years. A statistically significant age difference was observed between influenza A and influenza B infections, with median ages of 66 (IQR: 41–80) and 28 (IQR: 7–41) years, respectively (*p* < 0.001). Temporal trends in weekly case and control numbers are presented in Figure 1.

**TABLE 1:** Characteristics of cases and controls included in the study by vaccine status, 2024/25 influenza season in Northern Ireland. The five HSC Trusts included in the analysis were: Belfast (BHSCT), South Eastern (SEHSCT), Southern (SHSCT), Northern (NHSCT) and Western (WHSCT). Vaccine types: adjuvanted quadrivalent influenza vaccines (aQIV), live attenuated influenza vaccines (LAIV), cell‐based quadrivalent influenza vaccines (QIVc) and egg‐based quadrivalent influenza vaccines (QIVe).

	Controls		Cases		Overall	
	Unvaccinated (*N* = 7638)	Vaccinated (*N* = 5471)	Unvaccinated (*N* = 1383)	Vaccinated (*N* = 641)	Unvaccinated (*N* = 9021)	Vaccinated (*N* = 6112)
Age group						
2–17	1268 (16.6%)	752 (13.7%)	277 (20.0%)	86 (13.4%)	1545 (17.1%)	838 (13.7%)
18–64	3122 (40.9%)	846 (15.5%)	594 (43.0%)	105 (16.4%)	3716 (41.2%)	951 (15.6%)
65+	3248 (42.5%)	3873 (70.8%)	512 (37.0%)	450 (70.2%)	3760 (41.7%)	4323 (70.7%)
Sex						
Female	4135 (54.1%)	2772 (50.7%)	803 (58.1%)	313 (48.8%)	4938 (54.7%)	3085 (50.5%)
Male	3503 (45.9%)	2699 (49.3%)	580 (41.9%)	328 (51.2%)	4083 (45.3%)	3027 (49.5%)
Weeks since vaccination						
No vaccine	7638 (100%)	0 (0%)	1383 (100%)	0 (0%)	9021 (100%)	0 (0%)
16 + weeks	0 (0%)	1742 (31.8%)	0 (0%)	53 (8.3%)	0 (0%)	1795 (29.4%)
2–8 weeks	0 (0%)	1476 (27.0%)	0 (0%)	200 (31.2%)	0 (0%)	1676 (27.4%)
9–16 weeks	0 (0%)	2253 (41.2%)	0 (0%)	388 (60.5%)	0 (0%)	2641 (43.2%)
HSC Trust						
BHSCT	1827 (23.9%)	1192 (21.8%)	339 (24.5%)	146 (22.8%)	2166 (24.0%)	1338 (21.9%)
NHSCT	2036 (26.7%)	1545 (28.2%)	435 (31.5%)	221 (34.5%)	2471 (27.4%)	1766 (28.9%)
SEHSCT	1857 (24.3%)	1568 (28.7%)	297 (21.5%)	164 (25.6%)	2154 (23.9%)	1732 (28.3%)
SHSCT	1093 (14.3%)	704 (12.9%)	169 (12.2%)	60 (9.4%)	1262 (14.0%)	764 (12.5%)
WHSCT	789 (10.3%)	453 (8.3%)	140 (10.1%)	46 (7.2%)	929 (10.3%)	499 (8.2%)
Unknown	36 (0.5%)	9 (0.2%)	3 (0.2%)	4 (0.6%)	39 (0.4%)	13 (0.2%)

Vaccine type distribution reflected age‐based recommendations: the LAIV was predominantly administered to children aged 2–17 years; the QIVc was most commonly used in adults aged 18–64 years; and the adjuvanted egg‐based vaccine (aQIV) was primarily given to those aged 65 years and older. Among controls, the proportions of each vaccine type administered within each age group were as follows: in those aged 2–17 years, 96.3% (*n* = 724) received LAIV and 3.7% (*n* = 28) QIVc; in those aged 18–64 years, 94.7% (*n* = 801) received QIVc and 5.2% (*n* = 44) received aQIV; and in those aged ≥ 65 years, 97.6% (*n* = 3780) received aQIV and 2.4% (*n* = 93) received QIVc.

### 3.2 VE

**FIGURE 2:**
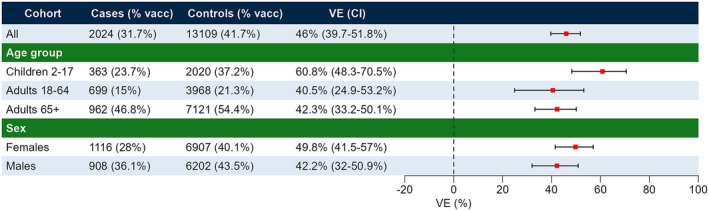
Vaccine effectiveness against any laboratory‐confirmed influenza, stratified by age group and sex.

VE against any laboratory‐confirmed influenza among all ages was 46.0% (95% CI: 39.7%–51.8%) (Figure 2). VE was highest among children aged 2–17 years at 60.8% (95% CI: 48.3%–70.5%), compared to adults aged ≥ 65 years at 42.3% (95% CI: 33.2%–50.1%) (*p* = 0.046) and adults aged 18–64 years at 40.5% (95% CI: 24.9%–53.2%) (*p* = 0.11). VE differed by sex, with higher protection observed among females (49.8%, 95% CI: 41.5%–57.0%) compared with males (42.2%, 95% CI: 32.0%–50.9%) (*p* = 0.04).

**FIGURE 3:**
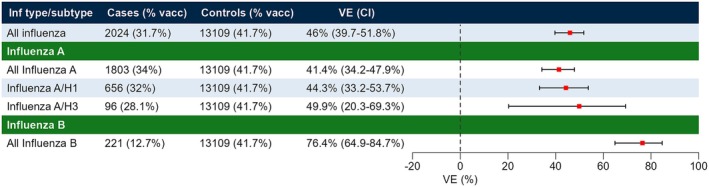
Vaccine effectiveness against laboratory‐confirmed influenza A and B, including influenza A subtypes A(H1) and A(H3).

Against influenza A infection, VE against any laboratory‐confirmed influenza‐associated hospitalisation was 41.4% (95% CI: 34.2%–47.9%) across all age groups (Figure 3). VE against influenza A(H1)‐associated hospital admissions was 44.3% (95% CI: 33.2%–53.7%), whereas VE against influenza A(H3)‐associated hospital admissions was 49.9% (95% CI: 20.3%–69.3%). In contrast, VE against influenza B was substantially higher than influenza A, with VE of 76.4% (95% CI: 64.9%–84.7%) (*p* < 0.0001).

**FIGURE 4:**
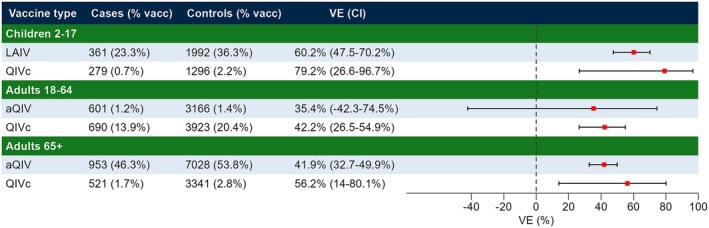
Vaccine effectiveness against laboratory‐confirmed influenza by vaccine type, stratified by age group.

We did not observe statistically significant differences in VE by vaccine type for children, adults aged 18–64 years or adults aged 65 + years (Figure 4). Among children aged 2–17 years, VE was 60.2% (95% CI: 47.5%–70.2%) for LAIV and QIVc at 79.2% (95% CI: 26.6%–96.7%) (*p* = 0.62). Among adults aged 18–64 years, VE was 35.4% (95% CI: −42.3%–74.5%) for aQIV and 42.2% (95% CI: 26.5%–54.9%) for QIVc (*p* = 0.97). In adults aged ≥ 65 years, VE was 41.9% (95% CI: 32.7%–49.9%) for aQIV and 56.2% (95% CI: 14.0%–80.1%) for QIVc (*p* = 0.83).

**FIGURE 5:**
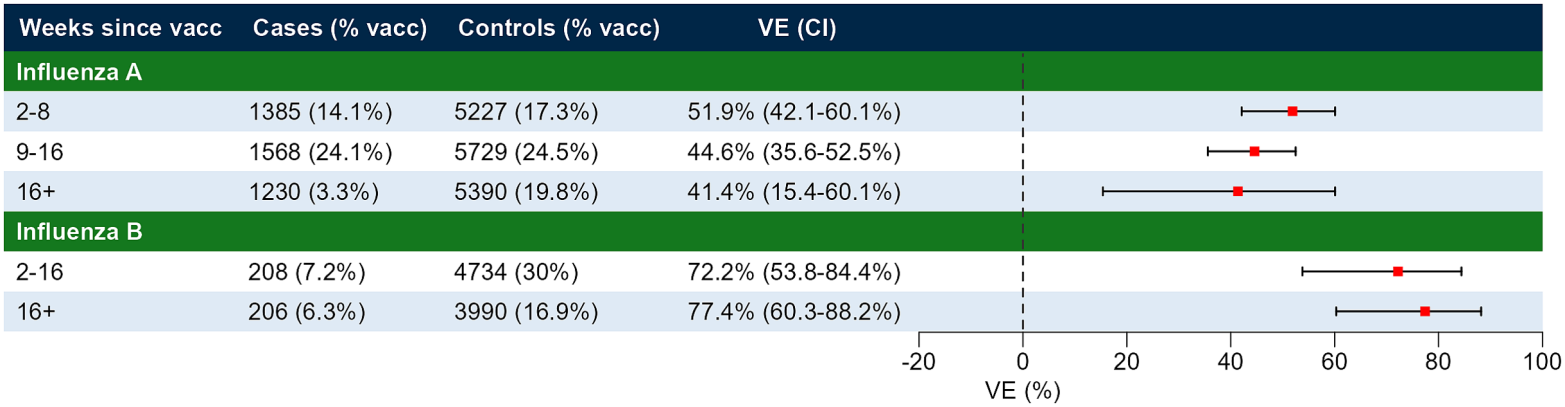
Vaccine effectiveness against laboratory‐confirmed influenza by time since vaccination for influenza A and influenza B.

To assess the duration of vaccine‐induced protection, VE was also estimated by time since vaccination (Figure 5). For influenza A, VE was highest within 2–8 weeks post‐vaccination (51.9%, 95% CI: 42.1%–60.1%) and was lower at 9–16 weeks (44.6%, 95% CI: 35.6%–52.5%) and ≥ 16 weeks (41.4%, 95% CI: 15.4%–60.1%), although these differences were not statistically significant (*p* = 0.22 and *p* = 0.80, respectively). VE was 72.2% (95% CI: 53.8%–84.4%) at 2–16 weeks and 77.4% (95% CI: 60.3%–88.2%) at ≥ 16 weeks, with no evidence of a significant difference in VE between these two periods (*p* = 0.07).

### 3.3 Sensitivity Analyses

Several sensitivity analyses were undertaken. First, individuals vaccinated 7–14 days prior to testing were included in an additional analysis to explore potential early vaccine effects during partial immune development; VE estimates were comparable to those from the primary analysis (Figure S1). Second, to minimise potential selection bias, individuals who tested positive for SARS‐CoV‐2 (*n* = 295) were retained within the control group. VE estimates for all influenza types differed by only 0.5 percentage points compared with the main analysis, with differences of 0.1, 0.4 and 1.0 percentage points among children, adults aged 18–64 years and adults aged ≥ 65 years, respectively (Figure S2), indicating minimal impact on the results. Estimates also differed by no more than 1.5 percentage points across vaccine type, influenza type/subtype and time since vaccination (Figures S3–S5). Third, Firth's penalised logistic regression was applied to reduce small‐sample bias in subgroup analyses, particularly for influenza A(H1), A(H3) and influenza B. VE estimates obtained using this approach were comparable to those from standard logistic regression (Figure S6).

Restricting the analysis to emergency admissions included 12,566 eligible patients (83% of all admissions), of whom 1746 (13.9%) tested positive for influenza; 1569 (89.9%) had influenza A and 177 (10.1%) had influenza B. VE estimates were broadly consistent with those from the primary analysis. VE against any influenza was 47.4% (95% CI: 40.8%–53.4%; Figure S7). VE was highest among children aged 2–17 years (58.2%, 95% CI: 44.3%–68.9%) compared to adults aged ≥ 65 years (44.3%, 95% CI: 35.0%–52.3%; *p* = 0.13) and those aged 18–64 years (42.1%, 95% CI: 24.4%–56.2%; *p* = 0.26). VE against influenza A was 43.3% (95% CI: 35.8%–50.0%). By subtype, VE was 47.4% (95% CI: 35.5%–57.2%) against A(H1) and 64.1% (95% CI: 38.1%–80.0%) against A(H3); the latter estimate was higher than that observed in the primary analysis, although confidence intervals overlapped. VE against influenza B remained higher than against influenza A (75.9%, 95% CI: 62.6%–85.1%; *p* < 0.0001; Figure S8).

VE against influenza A was 53.8% (95% CI: 43.8%–62.2%) within 2–8 weeks of vaccination, 46.5% (95% CI: 37.0%–54.7%) at 9–16 weeks (*p* = 0.3) and 39.8% (95% CI: 11.1%–60.1%) at ≥ 16 weeks (*p* = 0.73) (Figure S9). For influenza B, VE was estimated at 72.6% (95% CI: 52.0%–85.7%) at 2–16 weeks and 77.3% (95% CI: 58.1%–88.8%) at ≥ 16 weeks (*p* = 0.06). VE by vaccine type was similar to the primary analysis, although QIVc appeared to provide greater protection in children (87.8% vs. 79.2%) (Figure S10).

## 4 Discussion

This evaluation of the 2024/25 seasonal influenza vaccination programme in NI demonstrated protective effects against hospitalisation due to both influenza A and influenza B across age groups. The findings align with interim VE estimates reported in other European countries [7, 8, 10, 11].

VE estimates for the 2024/25 influenza season were broadly consistent with those observed in NI during 2023/24, with moderate protection against influenza‐associated hospitalisation overall and higher effectiveness in children than in adults [19]. VE against any laboratory‐confirmed influenza was similar across seasons (46.0% in 2024/25 and 47.5% in 2023/24), with consistently higher VE among children aged 2–17 years (60.8% in 2024/25 and 65.2% in 2023/24) and lower protection among adults aged 65 years and older (42.3% in 2024/25 and 39.5% in 2023/24). In both seasons, VE against influenza A was moderate and lowest among older adults, whereas VE against influenza B was substantially higher. Although VE against influenza B was lower in 2024/25 than in 2023/24 (76.4% vs. 87.2%), protection remained strong and exceeded that observed for influenza A in both seasons. In 2024/25, VE against influenza A declined progressively with increasing time since vaccination, while remaining protective beyond 4 months. A similar pattern was observed in 2023/24, with the highest VE within 2–8 weeks post‐vaccination and reduced effectiveness thereafter. Although waning was estimated for influenza A and B combined in 2023/24 and separately by virus type in 2024/25, this methodological difference is unlikely to materially affect comparability, as influenza A accounted for 98% of laboratory‐confirmed cases in 2023/24. Our findings should also be interpreted in the context of NI's use of age‐specific enhanced influenza vaccines, including LAIV for children and adjuvanted or cell‐based vaccines for eligible adult groups. The continued use of these enhanced formulations may have contributed to the sustained significant protection observed across seasons, particularly among children.

VE estimates in NI were broadly comparable with those reported in other settings in 2024/25. A European multi‐country study reported VE ranging from 34%–60% in secondary care, whereas studies from the United States showed moderate VE in outpatient settings and higher protection against influenza‐associated hospitalisation, particularly among children [8, 9, 10]. End‐of‐season analyses from France similarly reported VE of around 44%, with reduced effectiveness among adults aged 65 years and older, especially against influenza A (23%; 95% CI: 13%–32%) compared with influenza B (57%; 95% CI: 35%–72%) [20]. Collectively, these findings align closely with our estimates and reinforce the consistency of moderate VE and reduced protection in older populations across diverse healthcare settings.

The pattern of higher effectiveness among children compared with adults aligns with other TND studies and likely reflects differences in immune response, prior exposure history and underlying health status [8, 9, 21]. In Hong Kong, VE against hospitalisation among children was estimated at 64.8% (95% CI: 38.1%–80.3%) for influenza A(H1N1) and 59.9% (95% CI: −42.0%–89.6%) for influenza A(H3N2) [21]. VE was higher against influenza B than influenza A, consistent with reports from European multi‐country networks and national surveillance systems [8, 20]. These subtype‐specific differences are likely influenced by antigenic match, pre‐existing immunity and patterns of viral circulation.

This study draws on pseudonymised, linked, individual‐level data from multiple health system sources across NI. The VMS captures influenza vaccinations delivered through general practice, community pharmacies, HSC Trusts and school‐based programmes, providing near‐complete coverage of vaccination among eligible individuals. Laboratory confirmation of influenza was obtained from systems that rely on regional laboratory information management systems for the Regional Virology Laboratory and all HSC Trust point of care testing, enabling full regional surveillance. Although deterministic linkage using a unique patient identifier is expected to minimise linkage errors, inaccurate recording of identifiers within source datasets may have resulted in a small number of missed linkages.

Several limitations should be noted. Small numbers in several age‐ and subtype‐specific subgroups resulted in wider confidence intervals and reduced precision of some VE estimates. However, sensitivity analyses using Firth's penalised logistic regression produced similar findings, suggesting that small‐sample and sparse‐data bias had minimal impact on the overall conclusions. Residual confounding due to unmeasured factors, such as underlying health conditions and previous vaccination history, cannot be ruled out. As individuals with underlying medical conditions are more likely to be eligible for and receive influenza vaccination, failure to adjust for these factors may have biased VE estimates. For adults aged 18–64 years, eligibility for vaccination could not be determined. Though the test‐negative design aims to control for this, there may remain some potential for selection bias if vaccinated adults were more likely to have comorbidities that conferred eligibility, which could in turn lead to underestimation of VE in this age group. In addition, the proportion of vaccinated individuals among test‐negative controls differed across HSC Trusts. Although HSC Trust was included as an adjustment variable in all analyses, these differences may reflect variation in the demographic and clinical characteristics of patients undergoing influenza testing, healthcare utilisation patterns, or local testing practices. Consequently, some residual confounding due to unmeasured Trust‐level factors cannot be excluded. Finally, although influenza testing was undertaken through a clinician‐directed diagnostic pathway for suspected acute respiratory infection, presenting symptoms and ICD‐10‐coded admission diagnoses were unavailable. We therefore could not retrospectively apply a standardised clinical case definition and residual selection bias related to heterogeneous testing indications cannot be excluded.

## 5 Conclusion

In this end‐of‐season evaluation, seasonal influenza vaccination provided significant protection at individual and population level, with moderate effectiveness, against laboratory‐confirmed influenza‐associated hospitalisation in NI during the 2024/25 season. VE varied by age, sex and virus type, with consistently higher protection in children and against influenza B than influenza A. Although VE against influenza A declined numerically with increasing time since vaccination, differences were not significant and protection was maintained beyond 4 months. These findings support the continued role of seasonal influenza vaccination in reducing severe influenza outcomes and highlight the value of linked routine surveillance data for monitoring vaccine performance.

## Author Contributions

**Magda Bucholc:** methodology, investigation, validation, formal analysis, writing – original draft. **Siobhán Murphy:** validation, writing – original draft. **Mark G. O'Doherty:** data curation, investigation, validation, writing – original draft. **Suzanne Wilton:** data curation, writing – review and editing. **Emma Dickson:** data curation, investigation, validation, writing – original draft. **Declan T. Bradley:** conceptualization, methodology, investigation, validation, writing – review and editing.

## Funding

This study was funded by the Public Health Agency.

## Ethics Statement

This work was defined as health surveillance, not research, in accordance with the UK Health Research Authority (HRA) and National Research Ethics Service (NRES) guidance (https://www.hra‐decisiontools.org.uk/research/docs/DefiningResearchTable_Oct2022.pdf). Research Ethics Approval was therefore not required or sought. The analysis was undertaken as part of the Public Health Agency's routine infectious disease surveillance programme, carried out under its statutory public health protection functions as defined in the Health and Social Care (Reform) Act (Northern Ireland) 2009. No new data were collected for this work and all analyses involved pseudonymised routine administrative healthcare data accessed under existing information governance agreements with the relevant data controllers. These activities were conducted in line with the Department of Health's Code of Practice on Protecting the Confidentiality of Service User Information.

## Conflicts of Interest

The authors declare no conflicts of interest.

## Supporting information


**Figure S1:** Vaccine effectiveness (VE) against laboratory‐confirmed influenza including partially vaccinated individuals (sample collected 7–14 days post vaccination). VE stratified by age group and sex. Abbreviations: CI, confidence interval.
**Figure S2:** Vaccine effectiveness (VE) against laboratory‐confirmed influenza including controls who tested positive for SARS‐CoV‐2. VE stratified by age group and sex. Abbreviations: CI, confidence interval.
**Figure S3:** Vaccine effectiveness (VE) against laboratory‐confirmed influenza including controls who tested positive for SARS‐CoV‐2. VE against laboratory‐confirmed influenza A and B, including influenza A subtypes A(H1) and A(H3). Error bars represent 95% confidence intervals (CI).
**Figure S4:** Vaccine effectiveness (VE) against laboratory‐confirmed influenza including controls who tested positive for SARS‐CoV‐2. VE by vaccine type, stratified by age group. Error bars represent 95% confidence intervals (CI).
**Figure S5:** Vaccine effectiveness (VE) against laboratory‐confirmed influenza including controls who tested positive for SARS‐CoV‐2. VE by time since vaccination for laboratory‐confirmed influenza A and influenza B. Error bars represent 95% confidence intervals (CI).
**Figure S6:** Vaccine effectiveness against all laboratory‐confirmed influenza, influenza A and B, including influenza A subtypes A(H1) and A(H3) estimated using Firth's penalised logistic regression models. Error bars represent 95% confidence intervals (CI).
**Figure S7:** Vaccine effectiveness against laboratory‐confirmed influenza by age group and sex among emergency admissions. Error bars represent 95% confidence intervals (CI).
**Figure S8:** Vaccine effectiveness against laboratory‐confirmed influenza A, A subtypes and influenza B among emergency admissions. Error bars represent 95% confidence intervals (CI).
**Figure S9:** Vaccine effectiveness by time since vaccination among emergency admissions. Error bars represent 95% confidence intervals (CI).
**Figure S10:** Vaccine effectiveness by influenza vaccine type among emergency admissions. Error bars represent 95% confidence intervals (CI).

## Data Availability

Data supporting this study cannot be made available due to ethical and legal reasons.
