# Supplementary material for: Effectiveness of Influenza Vaccines and Duration of Protection Against Hospitalisation During the 2024/25 Season in Northern Ireland, UK
Source: Influenza Other Respir Viruses. 2026 Aug 6;20(8):e70303. doi: 10.1111/irv.70303 (PMC13446018; doi:10.1111/irv.70303)
Supplement: Supplementary file 1 [file IRV-20-e70303-s001.docx]

**Supplementary material**

**Figure S1.** Vaccine effectiveness (VE) against laboratory-confirmed influenza including partially vaccinated individuals (sample collected 7-14 days post vaccination). VE stratified by age group, and sex. Abbreviations: CI, confidence interval.

**Figure S2.** Vaccine effectiveness (VE) against laboratory-confirmed influenza including controls who tested positive for SARS-CoV-2. VE stratified by age group, and sex. Abbreviations: CI, confidence interval.

**Figure S3.** Vaccine effectiveness (VE) against laboratory-confirmed influenza including controls who tested positive for SARS-CoV-2. VE against laboratory-confirmed influenza A and B, including influenza A subtypes A(H1) and A(H3). Error bars represent 95% confidence intervals (CI).

**Figure S4.** Vaccine effectiveness (VE) against laboratory-confirmed influenza including controls who tested positive for SARS-CoV-2. VE by vaccine type, stratified by age group. Error bars represent 95% confidence intervals (CI).

**Figure S5.** Vaccine effectiveness (VE) against laboratory-confirmed influenza including controls who tested positive for SARS-CoV-2. VE by time since vaccination for laboratory-confirmed influenza A and influenza B. Error bars represent 95% confidence intervals (CI).

**Figure S6.** Vaccine effectiveness against all laboratory-confirmed influenza, influenza A and B, including influenza A subtypes A(H1) and A(H3) estimated using Firth’s penalised logistic regression models. Error bars represent 95% confidence intervals (CI).

**Figure S7.** Vaccine effectiveness against laboratory-confirmed influenza by age group and sex among emergency admissions. Error bars represent 95% confidence intervals (CI).

**Figure S8.** Vaccine effectiveness against laboratory-confirmed influenza A, A subtypes, and influenza B among emergency admissions. Error bars represent 95% confidence intervals (CI).

**Figure S9.** Vaccine effectiveness by time since vaccination among emergency admissions. Error bars represent 95% confidence intervals (CI).

**Figure S10.** Vaccine effectiveness by influenza vaccine type among emergency admissions. Error bars represent 95% confidence intervals (CI).
